# Impact of Circular Stapler Diameter on Anastomotic Leakage in Left-Sided Colorectal Cancer: A Retrospective Single-Center Case–Control Analysis

**DOI:** 10.3390/medicina61122231

**Published:** 2025-12-17

**Authors:** Ertuğrul Gazi Alkurt, Mert Yiğit Akdoğan, Mehmet Berksun Tutan, Bahadır Kartal, Veysel Barış Turhan

**Affiliations:** 1 Department of General Surgery, Faculty of Medicine, Hitit University, 19040 Corum, Türkiye; myigitakdogan@gmail.com (M.Y.A.); drbaristurhan@hotmail.com (V.B.T.); 2 Department of General Surgery, Alaca State Hospital, 19600 Corum, Türkiye; mbtutan@gmail.com; 3 Department of General Surgery, Erol Olçok Training and Research Hospital, 19040 Corum, Türkiye; dr.bkartal@hotmail.com

**Keywords:** colorectal cancer, anastomotic leakage, stapler diameter, circular stapler, serum protein, nutritional status

## Abstract

*Background and Objectives*: Anastomotic leakage (AL) is a major complication following sphincter-preserving surgeries for left-sided colorectal cancer. In this study, we aimed to evaluate the association between circular stapler diameter and the risk of AL. As a secondary objective, we investigated whether preoperative serum protein levels were associated with leakage development. *Materials and Methods*: We conducted a retrospective case–control study including 99 patients who underwent elective colorectal surgery with stapled anastomosis for left-sided colorectal cancer between January 2020 and May 2024. A total of 99 patients were included (60.6% male), with a mean age of 66.1 ± 10.7 years. The patients were categorized into small (≤29 mm) and large (≥30 mm) stapler groups. Demographic, clinical, and laboratory variables were collected. Anastomotic leakage was defined as an International Study Group of Rectal Cancer (ISREC) Grade B or C leak requiring intervention. Univariate analyses and multivariate logistic regression analyses were performed, and results were reported as odds ratios (ORs) with 95% confidence intervals (CIs). A STROBE-compliant flow diagram was prepared. *Results*: Anastomotic leakage occurred in 10 patients (10.1%), and leakage rates were not significantly different between stapler-size groups (≤29 mm: 10.9% vs. ≥30 mm: 7.5%, *p* = 0.365). In multivariate analysis, stapler size was not independently associated with leakage (OR 1.68, 95% CI 0.40–6.97, *p* = 0.480). Lower preoperative serum protein levels were identified as the only independent predictor of leakage (OR 0.28, 95% CI 0.10–0.74, *p* = 0.011). Postoperative hospital stay was significantly longer for patients with leakage (median 17 vs. 7 days, *p* < 0.001). *Conclusions*: We found no significant associations between circular stapler diameter and anastomotic leakage in left-sided colorectal cancer surgery. Conversely, low serum protein levels were independently associated with increased leakage risk, highlighting the importance of preoperative nutritional assessment. Given the retrospective design, small number of leakage cases, and unmeasured confounders, these findings should be interpreted with caution. Further multicenter, prospective studies should be conducted to clarify the influence of stapler size and patient-related factors on anastomotic outcomes.

## 1. Introduction

Colorectal cancer (CRC) is one of the most frequently diagnosed malignancies worldwide, and it is associated with high rates of morbidity and mortality. In particular, sphincter-preserving surgical approaches such as anterior resection and low anterior resection have become standard procedures for left-sided colon and rectal tumors. Intestinal anastomoses performed during these operations are critical determinants of surgical success. Anastomotic leakage (AL) is one of the most serious complications of such procedures, with reported incidence rates ranging from 1% to 28% in the literature. This complication significantly increases the length of hospital stay, morbidity, reoperation rates, and even mortality [1,2,3]. Consequently, technical and technological strategies aimed at enhancing anastomotic safety remain a major focus in colorectal surgery.

Circular staplers are widely used for colorectal anastomoses, and they are available in various diameters. In clinical practice, stapler size is typically selected based on distal bowel diameter, tumor location, and the surgeon’s technical preferences. However, the impact of stapler diameter on the risk of anastomotic leakage remains controversial in the literature. For example, Jiang et al. reported that the use of a 32 mm stapler was associated with a significantly higher rate of leakage (AL 13.4% vs. 2.9%, *p* = 0.019) [1]. In contrast, Nagaoka et al. found no significant associations between stapler diameter and AL, suggesting that stapler size alone may not be a determining factor [4]. Previous studies have commonly categorized stapler sizes into groups of ≤29 mm versus ≥30 mm based on both clinical practice and published evidence indicating different risks of anastomotic complications [1,5,6]. Thus, our study adopted the same cut-off in order to facilitate comparability with the literature.

It should be noted that left-sided colonic anastomoses and rectal anastomoses differ substantially in terms of anatomical location (intraperitoneal vs. extraperitoneal), vascular perfusion, pelvic anatomy, stapling difficulty, and the frequency of protective stoma creation. These distinctions may influence anastomotic leakage risk, and subgroup analyses separating colonic and rectal anastomoses are often recommended to clarify such associations.

Mechanistically, stapler size may affect local perfusion, tissue compression, and the degree of lumen mismatch at the anastomotic site, which could in turn influence the risk of leakage. However, evidence on this topic remains inconsistent, underscoring the need for further investigation.

The primary aim of this study was to investigate whether there is an association between circular stapler diameter and the occurrence of anastomotic leakage in patients undergoing surgery for left-sided colorectal cancer. Our primary hypothesis was that the use of smaller stapler diameters (≤29 mm) may increase the risk of leakage. In contrast to previous versions of this manuscript, the role of nutritional status (e.g., serum protein levels) was not considered a primary objective but was instead evaluated as a secondary factor potentially associated with leakage. By focusing on stapler diameter while also exploring relevant clinical parameters, we aimed to provide a comprehensive assessment of risk factors for AL in this patient population.

## 2. Materials and Methods

This retrospective case–control study was conducted at Hitit University Faculty of Medicine, Çorum, Turkey, and included patients who underwent elective surgery for left-sided colon or rectal cancer between January 2020 and May 2024. All patient data were obtained from the institutional electronic medical record system, operative reports, and discharge summaries.

### 2.1. Inclusion and Exclusion Criteria

To ensure methodological clarity and reproducibility, the study population was de-fined using the following eligibility criteria:

#### 2.1.1. Inclusion Criteria

- Patients aged ≥18 years
- Histopathologically confirmed adenocarcinoma located in the descending colon, sigmoid colon, or rectum
- Elective surgical intervention
- Construction of a stapled colorectal anastomosis using a circular stapler
- Availability of complete demographic, clinical, laboratory, operative, and postoperative data

#### 2.1.2. Exclusion Criteria

- Patients aged <18 years
- Emergency colorectal procedures
- Surgery performed for benign conditions (e.g., diverticular disease, inflammatory bowel disease)
- Use of manual (hand-sewn) anastomosis instead of a circular stapler
- Radiologically detected but asymptomatic ISREC Grade A leaks
- Incomplete or missing medical records and cases with insufficient postoperative follow-up
- Procedures lacking clear documentation of anastomotic configuration

The cut-off for stapler size (≤29 mm vs. ≥30 mm) was selected based on previously published studies indicating a potential difference in leakage risk across these groups [1,5,6]. All circular staplers used during the study period were single-use manual devices. Power-assisted or automatic circular stapling systems were not available at our institution and therefore were not used.

Patient data were obtained retrospectively by reviewing the hospital information system, operative reports, and discharge summaries. A standardized data collection form was used for each patient, and the collected information was categorized under four main domains. The demographic data collected included age, sex, and comorbidities (e.g., diabetes mellitus, hypertension, and chronic kidney disease), and the preoperative assessments included the ASA (American Society of Anesthesiologists) physical status score, hemoglobin levels, total serum protein, serum albumin, and white blood cell counts. These laboratory values were obtained from tests performed within 72 h prior to surgery. The surgical variables included tumor location (descending colon, sigmoid colon, or rectum), type of surgical procedure (anterior resection, low anterior resection, or left hemicolectomy), and stapler diameter. Stapler size was categorized as ≤29 mm (the small-diameter group) and ≥30 mm (the large-diameter group). All staplers used were single-use, single-fire devices. The specific brands and staple-row configurations could not be uniformly retrieved in this retrospective review and were therefore excluded from the analysis; this was acknowledged as a limitation. Whether a protective loop ileostomy was performed was recorded in the surgical notes. Oncologic and perioperative variables such as TNM stage, neoadjuvant therapy, and surgical approach (laparoscopic vs. open) were not available in the dataset and therefore excluded from the analysis. This absence is addressed in the section on limitations.

In the postoperative period, anastomotic leakage was diagnosed based on clinical findings such as fever, abdominal pain, guarding, leukocytosis, and fecal discharge from drains, combined with evidence from contrast-enhanced abdominal computed tomography (CT) showing extraluminal leakage or pelvic collections or findings obtained during reoperation if applicable. Leaks were classified according to the ISREC (International Study Group of Rectal Cancer) grading system, and only clinically relevant Grade B and C leaks requiring intervention were included. Asymptomatic Grade A leaks identified only via imaging were excluded. All leakage diagnoses were confirmed by the treating surgical team according to standardized ISREC criteria.

All statistical analyses were performed using SPSS version 27.0 (IBM Corp., Armonk, NY, USA). Continuous variables were expressed as means ± standard deviation (SD) for normally distributed data (e.g., age and hemoglobin) and as medians (ranges) for non-normally distributed data (e.g., white blood cell count, total protein, albumin, and length of hospital stay). Categorical variables were presented as counts and percentages.

Univariate analyses were used to compare characteristics between patients with and without anastomotic leakage. The independent-samples t-test was used for normally distributed continuous variables, and the Mann–Whitney U test was applied for non-normally distributed variables. Categorical variables were compared using the Chi-square test.

Following univariate analyses, binary logistic regression was performed to identify independent predictors of anastomotic leakage. The dependent variable was the presence of leakage, while the independent variables included stapler size, age, hemoglobin level, serum protein level, gender, and use of a protective loop ileostomy. Results were reported as odds ratios (ORs) with 95% confidence intervals (CIs), regression coefficients, standard errors, Wald statistics, and *p*-values. Model calibration was assessed using the Hosmer–Lemeshow test. Multicollinearity was evaluated via variance inflation factor (VIF) analysis. A two-sided *p*-value < 0.05 was considered statistically significant.

## 3. Results

**Figure 1:**
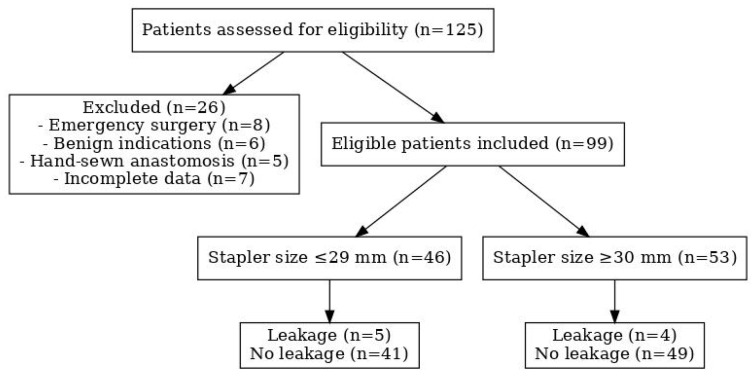
STROBE flow diagram.

A total of 99 patients who underwent surgery with anastomosis were included in this study; in 10 of these patients, anastomotic leakage occurred (10.1%). The STROBE-compliant flow diagram below (Figure 1) illustrates patient inclusion, exclusion, and final allocation.

The demographic, clinical, and perioperative characteristics of the patients with and without leakage are summarized in Table 1. There were no significant differences between the leakage and non-leakage groups in terms of gender (male: 77.78% vs. 59.55%, *p* = 0.521) and age (72.11 ± 9.48 vs. 65.85 ± 10.25 years, *p* = 0.443). The ASA score distribution did not differ significantly between the groups (*p* = 0.950). Laboratory values such as white blood cell count (WBC) (*p* = 0.519), hemoglobin levels (*p* = 0.964), and albumin (*p* = 0.074), were also similar between groups. Although not statistically significant in univariate analysis, patients with leakage had lower protein levels than those without leakage (median 6.5 vs. 6.7 g/dL, *p* = 0.061). Anastomotic leakage occurred in 10.9% of patients in the ≤29 mm stapler group and 7.5% of patients in the ≥30 mm group, and this difference was not statistically significant (*p* = 0.365).

Patient characteristics were also analyzed according to stapler-diameter groups (≤29 mm vs. ≥30 mm) (Table 1b). No significant differences were observed between groups in terms of age, sex distribution, ASA classification, hemoglobin, protein, albumin, WBC, or protective ileostomy rates. Anastomotic leakage occurred in 10.9% of patients in the ≤29 mm stapler group and 7.5% of patients in the ≥30 mm group, and this difference was not statistically significant (*p* = 0.365).

Postoperative hospitalization duration was significantly longer for patients with leakage compared to those without it (median 17 vs. 7 days, *p* < 0.001).

To further investigate the independent risk factors associated with the development of anastomotic leakage, a binary logistic regression analysis was conducted. Variables were selected for inclusion in the multivariate model based on clinical relevance and a univariate *p*-value threshold of <0.20. The final model included stapler size, age, hemoglobin concentration, serum protein level, gender, and the use of a protective loop ileostomy.

The results of the logistic regression analysis are presented in Table 2, which has been revised to include odds ratios (ORs) with 95% confidence intervals (CIs). Model calibration was verified using the Hosmer–Lemeshow test (*p* = 0.62), and no multicollinearity was detected (all VIF values < 2).

Among the parameters evaluated, only serum protein level emerged as a statistically significant independent predictor of anastomotic leakage. Specifically, lower serum protein levels were associated with a higher likelihood of leakage (OR: 0.28, 95% CI: 0.10–0.74, *p* = 0.011). Other variables, including stapler size (*p* = 0.480), age (*p* = 0.580), hemoglobin (*p* = 0.270), gender (*p* = 0.546), and use of a protective loop ileostomy (*p* = 0.110), did not demonstrate statistically significant associations with anastomotic leakage in the multivariate model.

Given the relatively small number of leakage cases (n = 10), the results should be interpreted with caution. Confidence intervals were wide for several predictors, reflecting limited statistical power.

## 4. Discussion

In this retrospective case–control study, the relationship between circular stapler diameter and the development of anastomotic leakage (AL) was investigated in patients undergoing surgery for left-sided colorectal cancer. When demographic and nutritional variables were considered, stapler diameter did not independently influence the risk of anastomotic leakage, suggesting that patient-related physiological factors have a greater impact on anastomotic integrity than stapler size. In our cohort, leakage occurred in 10.9% of patients in the ≤29 mm group compared with 7.5% in the ≥30 mm group; however, this difference did not reach statistical significance (*p* = 0.365). Although the rate of leakage was higher in the small-diameter stapler group (10.9%), this difference did not reach statistical significance (*p* = 0.365). Importantly, multivariate logistic regression analysis identified only low serum protein levels as an independent predictor of leakage (*p* = 0.011), whereas other variables, including stapler diameter, age, hemoglobin level, sex, and protective loop ileostomy, did not show statistically significant associations. These findings suggest that patient-related physiological parameters reflecting nutritional status may play a more prominent role in the development of anastomotic complications than stapler diameter alone.

The potential mechanisms by which stapler diameter could influence leakage include impaired blood perfusion at the anastomotic site due to excessive tissue compression, the technical difficulty of achieving even staple formation in narrow pelvic spaces, and mismatch between bowel lumen and stapler size leading to increased mechanical tension. Despite these plausible mechanisms, our findings—consistent with several recent reports—suggest that stapler size alone is not a decisive risk factor.

In our cohort, the leakage rate was 10.9% in the ≤29 mm group and 7.5% in the ≥30 mm group, though this was not statistically significant (*p* = 0.365). Similarly, in the study by Jiang et al., leakage occurred in 2.9% of patients treated with 29 mm staplers and in 13.4% of those treated with 32 mm staplers, indicating a higher risk associated with larger staplers [1]. Fiorillo et al., in a comprehensive meta-analysis, found that the use of 31/33 mm staplers were associated with a higher leakage rate compared to that for 28/29 mm staplers (OR −0.92; 95% CI −1.74 to −0.10; *p* = 0.02) [5]. In contrast, Behboudi et al. found no significant differences in leakage rates between 29 mm and 31 mm stapler groups [6], while Lee et al. reported no significant differences between 25 mm and 28–29 mm staplers [7]. Nagaoka et al. [4] also found no associations between stapler size and leakage, aligning closely with our results. These heterogeneous findings throughout the literature highlight that stapler diameter likely interacts with multiple factors, including patient anatomy, tumor location, operative technique, and surgeon experience, rather than acting as an isolated determinant.

In clinical practice, the choice of circular stapler diameter is guided by intraoperative evaluation of the proximal colonic lumen [8]. Surgeons typically select the smallest stapler that allows safe insertion without excessive stretching of the bowel wall. Overstretching the proximal colon over the anvil may impair microvascular perfusion, increase mechanical tension, and theoretically predispose to anastomotic failure. Although stapler sizes in our study were categorized as ≤29 mm and ≥30 mm, all staplers were selected based on the surgeon’s intraoperative assessment of bowel diameter. This individualized approach may partially explain the absence of a significant difference in leakage rates between stapler groups.

Our study identified low serum protein level as the only independent predictor of anastomotic leakage. This observation supports previous reports linking malnutrition with impaired anastomotic healing. No other preoperative demographic or clinical fac-tors—including age, sex, ASA classification, hemoglobin, albumin, or WBC—were associated with anastomotic leakage in either univariate or multivariate analyses. For example, Yamano et al. demonstrated that malnutrition was associated with increased risk of AL following rectal cancer surgery [9], and Xu and Kong also confirmed malnutrition-related factors as significant predictors [10]. While we did not define a specific cutoff value for serum protein, our findings underscore the importance of preoperative nutritional assessment and, where necessary, optimization before surgery.

Recent studies have suggested that serum butyrylcholinesterase levels may serve as a novel biomarker associated with postoperative infectious complications and surgical site infection severity following colorectal cancer surgery. Incorporating such biochemical markers into future models may improve the predictive performance for anastomotic leakage [11].

The limitations of this study must be acknowledged. First, its retrospective and single-center design introduces inherent risks of selection bias and limits generalizability. Second, the relatively small number of leakage cases (n = 10) reduced statistical power, leading to wide confidence intervals and limiting the stability of regression models. Third, we were unable to stratify the cohort by important clinical factors such as TNM staging, neoadjuvant therapy, and surgical approach (laparoscopic vs. open), all of which are known to influence leakage risk. Fourth, detailed technical information regarding stapler brand, staple height, closed leg length, and surgeon experience could not be consistently retrieved and were therefore excluded from the analysis. Fifth, no formal assessments of inter-rater reliability for leakage diagnosis were performed, although standardized ISREC criteria were applied. Another limitation is that only manual circular staplers were used in this cohort. Pow-er-assisted stapling systems, which may influence staple formation and anastomotic characteristics, were not available during the study period. Thus, potential differences between manual and powered circular staplers could not be assessed. Finally, the unequal case/control ratio and the absence of matching may have introduced imbalances between groups. These limitations are important sources of potential bias and imprecision, and readers should interpret the findings with caution.

Despite these limitations, this study contributes to the ongoing debate on the role of stapler diameter in colorectal surgery. Our findings suggest that stapler size alone should not be considered a decisive risk factor for anastomotic leakage. Instead, a more individualized approach—considering patient anatomy, tumor characteristics, and nutritional status—may be more appropriate.

From a clinical perspective, surgeons should avoid rigidly adhering to a single stapler size and instead prioritize patient-specific factors when planning anastomoses. From a research standpoint, prospective multicenter studies with standardized surgical protocols and larger sample sizes and incorporating oncologic variables are required in order to validate these findings and clarify the interplay between technical and patient-related factors.

## 5. Conclusions

In this retrospective study, circular stapler diameter was not significantly associated with anastomotic leakage in left-sided colorectal cancer surgery. In contrast, low preoperative serum protein level emerged as the only independent predictor of leakage, underscoring the relevance of nutritional status in anastomotic healing. These findings should be interpreted with caution due to the limited number of leakage events, and larger prospective studies are needed to confirm these results.

## Figures and Tables

**Table 1:** (**a**) Baseline demographic and clinical characteristics according to anastomotic leakage status. (**b**) Baseline characteristics according to stapler-size group.

**(a)**				
**Variables**	**All Patients (n = 99)**	**No Leakage (n = 89)**	**Leakage (n = 10)**	***p*** **-Value**
Gender (male, %)	60 (60.61%)	53 (59.55%)	7 (77.78%)	0.521
Age (years, mean ± SD)	66.13 ± 10.67	65.85 ± 10.25	72.11 ± 9.48	0.443
ASA I–II (%)	47 (47.47%)	43 (48.31%)	3 (33.33%)	
ASA III–IV (%)	52 (52.53%)	46 (51.69%)	6 (66.67%)	0.950
Hemoglobin (g/dL)	12.11 ± 1.82	12.10 ± 1.79	12.12 ± 2.31	0.964
Protein (g/dL)	6.6 (4.2–8.3)	6.7 (4.2–8.3)	6.5 (4.5–7.1)	0.061
Albumin (g/dL)	3.9 (1.8–4.8)	3.9 (1.8–4.8)	3.7 (2.5–4.2)	0.074
WBC (×10^3^/µL)	7.14 (2.43–19.65)	7.06 (2.43–19.65)	7.76 (4.96–9.83)	0.519
Stapler size ≤ 29 mm (%)	46 (46.46%)	40 (44.94%)	5 (55.56%)	0.365
Stapler size ≥ 30 mm (%)	53 (53.54%)	49 (55.06%)	4 (44.44%)	
Loop ileostomy (%)	67 (67.68%)	62 (69.66%)	4 (44.44%)	0.207
Hospital stays (days)	7 (4–36)	7 (4–36)	17 (7–35)	<0.001
**(b)**				
**Variables**	**≤29 mm (n = 46)**	**≥30 mm (n = 53)**	***p*** **-Value**	
Age (years, mean ± SD)	66.9 ± 10.5	65.5 ± 10.8	0.421	
Gender (Male, %)	28 (60.9%)	32 (60.4%)	0.962	
ASA I–II (%)	22 (47.8%)	25 (47.2%)	0.945	
ASA III–IV (%)	24 (52.2%)	28 (52.8%)		
Hemoglobin (g/dL)	12.0 ± 1.9	12.2 ± 1.8	0.627	
Protein (g/dL)	6.6 (4.4–7.9)	6.7 (4.2–8.3)	0.573	
Albumin (g/dL)	3.8 (2.0–4.7)	3.9 (1.8–4.8)	0.411	
WBC (×10^3^/µL)	7.2 (2.8–17.5)	7.1 (2.4–19.6)	0.804	
Leakage (%)	5 (10.9%)	4 (7.5%)	0.365	
Loop ileostomy (%)	30 (65.2%)	37 (69.8%)	0.624	
Hospital stays (days)	8 (4–35)	7 (4–36)	0.532	

Table 1a presents the univariate analysis of all demographic, clinical, and perioperative variables evaluated for their association with anastomotic leakage. Variables with *p* < 0.20, along with clinically relevant factors, were subsequently included in the multivariate logistic regression model.

**Table 2:** Multivariate logistic regression analysis for predictors of anastomotic leakage.

Variables	Estimate	SE	OR (95% CI)	Wald	*p*-Value
Stapler size (≤29 mm)	0.520	0.736	1.68 (0.40–6.97)	0.499	0.480
Age	0.020	0.036	1.02 (0.95–1.09)	0.306	0.580
Hemoglobin	0.265	0.240	1.30 (0.82–2.07)	1.219	0.270
Protein	–1.283	0.504	0.28 (0.10–0.74)	6.494	0.011
Protective loop ileostomy	–1.210	0.756	0.30 (0.07–1.22)	2.561	0.110
Gender (female)	–0.526	0.871	0.59 (0.11–3.13)	0.364	0.546

## Data Availability

The dataset used in this article is available from the corresponding author on reasonable request.
